# Prevention of Malaria Resurgence in Greece through the Association of Mass Drug Administration (MDA) to Immigrants from Malaria-Endemic Regions and Standard Control Measures

**DOI:** 10.1371/journal.pntd.0004215

**Published:** 2015-11-19

**Authors:** Maria Tseroni, Agoritsa Baka, Christina Kapizioni, Georges Snounou, Sotirios Tsiodras, Maria Charvalakou, Maria Georgitsou, Maria Panoutsakou, Ioanna Psinaki, Maria Tsoromokou, George Karakitsos, Danai Pervanidou, Annita Vakali, Varvara Mouchtouri, Theano Georgakopoulou, Zissis Mamuris, Nikos Papadopoulos, George Koliopoulos, Evangelos Badieritakis, Vasilis Diamantopoulos, Athanasios Tsakris, Jenny Kremastinou, Christos Hadjichristodoulou, MALWEST Project

**Affiliations:** 1 Department of Hygiene and Epidemiology, Faculty of Medicine, University of Thessaly, Larissa, Greece; 2 Hellenic Center for Disease Control & Prevention (HCDCP), Athens, Greece; 3 Sorbonne Universités, Paris, France; 4 Centre d’Immunologie et de Maladies Infectieuses (CIMI)—Paris, Institut National de la Santé et de la Recherche Médicale (Inserm)–Centre National de la Recherche Scientifique (CNRS), Paris, France; 5 Department of Biochemistry and Biotechnology, University of Thessaly, Larissa, Greece; 6 Laboratory of Entomology and Agricultural Zoology, School of Agricultural Sciences, Department of Agriculture Crop Production and Rural Environment, University of Thessaly, Volos, Greece; 7 Benaki Phytopathological Institute (BPI), Athens, Greece; 8 Municipality of Evrotas, Laconia Regional Unit, Tripoli, Greece; 9 Department of Microbiology, Faculty of Medicine, National and Kapodistrian University of Athens, Athens, Greece; Johns Hopkins Bloomberg School of Public Health, UNITED STATES

## Abstract

Greece was declared malaria-free in 1974 after a long antimalarial fight. In 2011–2012, an outbreak of *P*. *vivax* malaria was reported in Evrotas, an agricultural area in Southern Greece, where a large number of immigrants from endemic countries live and work. A total of 46 locally acquired and 38 imported malaria cases were detected. Despite a significant decrease of the number of malaria cases in 2012, a mass drug administration (MDA) program was considered as an additional measure to prevent reestablishment of the disease in the area. During 2013 and 2014, a combination of 3-day chloroquine and 14-day primaquine treatment was administered under direct observation to immigrants living in the epicenter of the 2011 outbreak in Evrotas. Adverse events were managed and recorded on a daily basis. The control measures implemented since 2011 continued during the period of 2013–2014 as a part of a national integrated malaria control program that included active case detection (ACD), vector control measures and community education. The MDA program was started prior to the transmission periods (from May to December). One thousand ninety four (1094) immigrants successfully completed the treatment, corresponding to 87.3% coverage of the target population. A total of 688 adverse events were recorded in 397 (36.2%, 95% C.I.: 33.4–39.1) persons, the vast majority minor, predominantly dizziness and headache for chloroquine (284 events) and abdominal pain (85 events) for primaquine. A single case of primaquine-induced hemolysis was recorded in a person whose initial G6PD test proved incorrect. No malaria cases were recorded in Evrotas, Laconia, in 2013 and 2014, though three locally acquired malaria cases were recorded in other regions of Greece in 2013. Preventive antimalarial MDA to a high-risk population in a low transmission setting appears to have synergized with the usual antimalarial activities to achieve malaria elimination. This study suggests that judicious use of MDA can be a useful addition to the antimalarial armamentarium in areas threatened with the reintroduction of the disease.

## Author Summary

Greece was declared malaria-free in the year 1974 after a long antimalarial fight. In 2011–2012, a number of malaria cases reported in Evrotas, Laconia, in Southern Greece, where a large number of immigrants from malaria-endemic countries live and work. A total of 84 malaria cases, both in immigrants (38 cases) and in Greeks (46 cases), were detected. A number of malaria control measures were deployed in the area since 2011. Despite a decrease of the number of malaria cases in 2012, elimination could not be achieved, and thus antimalarial MDA was considered as an additional measure to prevent the reestablishment of the disease. During 2013 and 2014, a combination of two drugs was administered under direct observation to all immigrants in the epicenter of the 2011 Evrotas outbreak. The antimalarial MDA program was started on July-August and was successfully completed by 1094 immigrants (87.3% coverage). No serious adverse events were recorded except one case of primaquine-induced hemolysis due to false G6PD test result. In 2013 and 2014, no malaria cases were recorded in Evrotas, Laconia. This study suggests that careful use of supervised antimalarial MDA is a useful addition to the antimalarial control measures in areas threatened with the reintroduction of the disease.

## Introduction

**Fig 1:**
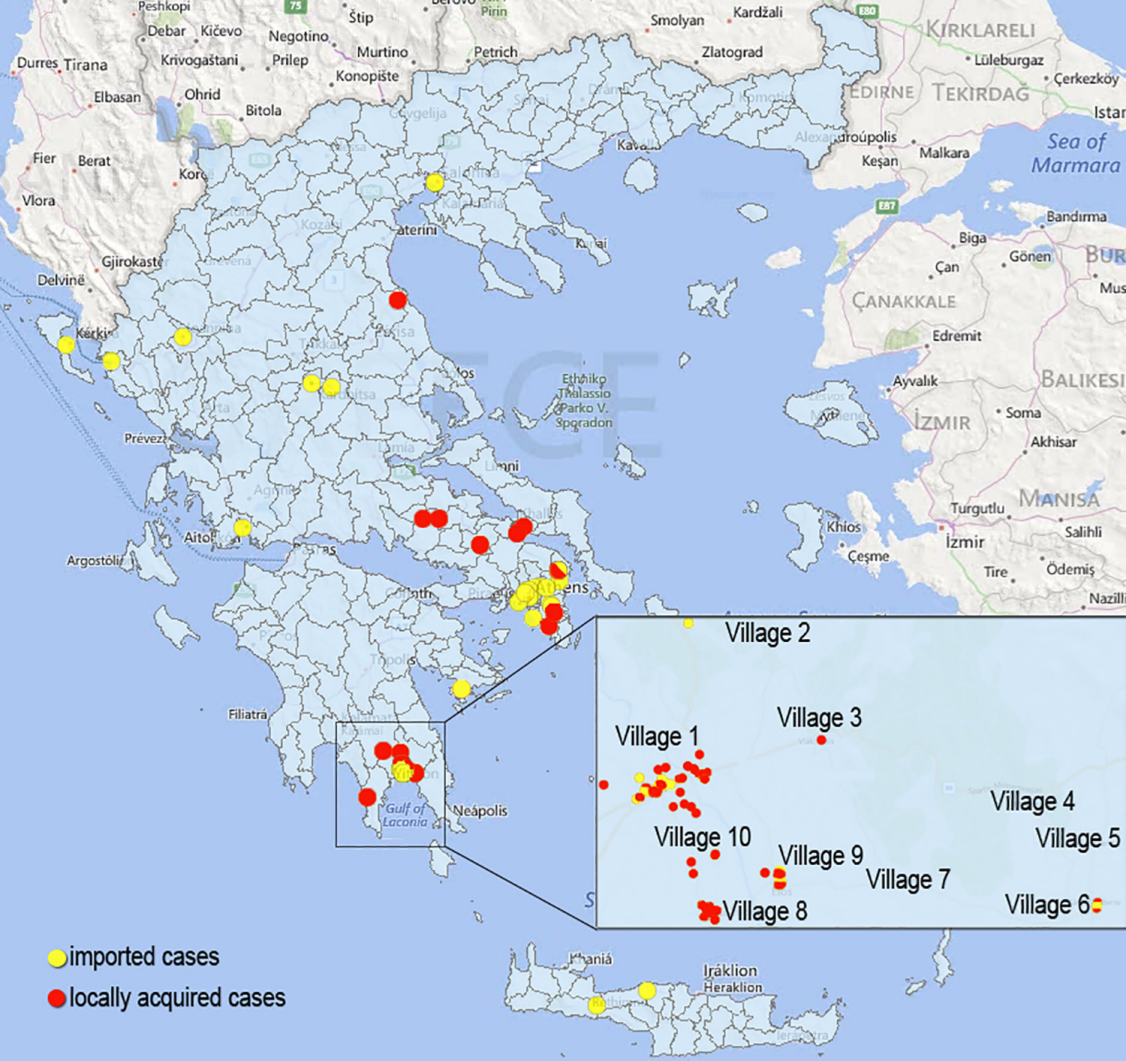
Geographic distribution of malaria cases (imported and locally acquired) in Greece 2011–2014 focusing on Evrotas, Laconia.

Greece was declared free of malaria in the year 1974 after many years of intense public health efforts [1]. Since then, a small number of imported cases have been reported annually [2], though rare sporadic cases raising the possibility of local transmission were also detected from time to time [3]. In 2009 and 2010 cases of *P*. *vivax* malaria (n = 6 and n = 1, respectively) most probably locally acquired were recorded in the agricultural area of Evrotas, Laconia in Peloponnese in Southern Greece [4]. In 2011, an outbreak of 36 confirmed locally acquired *P*. *vivax* cases were recorded in Greek citizens with no history of travel, and 21 imported cases in immigrants from non-endemic countries in the same area [5]. After the 2011 outbreak, a multidisciplinary strategy with a variety of intensive response activities, was adopted and implemented in Evrotas [1]. Nonetheless, in 2012, 10 locally acquired cases and 17 imported cases were again recorded [5]. The geographic distribution of malaria cases (imported and locally acquired) in Greece during 2011–2012 is shown in Fig 1.

Transmission was probably a result of the free contact between the competent malaria vector *Anopheles sacharovi*, an anopheline species prevalent in Greece and in the area of Laconia, and the immigrants from *P*. *vivax*-endemic countries (principally Pakistan, Afghanistan and Bangladesh). The immigrants came to Evrotas to work as farm laborers and possibly were the source of gametocytes [1]. There was a real concern that the yearly occurrence of locally acquired cases presaged the reestablishment of malaria in an area that was historically a hotspot of malaria transmission in Greece [6].

Over the past decades malaria control and elimination strategies have led to remarkable progress. Increased resources combined with effective antimalarial drugs, vector control measures, community engagement and participation, as well as robust malaria surveillance, have led to an expansion of antimalaria interventions in many countries [7], where their efficacy was increased by optimizing the deployment strategies to different transmission settings [8]. In view of the apparent failure of the initial control measures to prevent the occurrence of further locally acquired cases in Evrotas in 2012, despite the significant decrease of malaria cases, it was decided to adopt a protocol for MDA strategy to prevent further malaria transmission. Moreover, based on microsatellite analysis of the 2011 malaria cases in Evrotas, a number of cases among immigrants, which were characterized as imported based on epidemiological criteria, were afterwards considered as locally acquired [9]. MDA strategy implies that the entire population of the area concerned is provided with a curative dose of antimalarial drug(s) without prior testing for the presence of malaria infection or symptoms [10]. The use of MDA in malaria control is currently somewhat controversial. Although MDA was at times used until the 1970’s when the World Health Organization (WHO) recommended its implementation only in “exceptional conditions when conventional control techniques have failed”, the approach was eventually abandoned due to disappointing results [11]. Basically, the decline in parasite incidence or prevalence and the associated reduced mortality and morbidity observed during the intervention did not persist thereafter [7,11–14], though the deployment of MDA in some isolated areas did result in the interruption of transmission [15,16].

Given that the recent locally acquired malaria outbreaks in Greece were principally recorded in Evrotas, Laconia, it seemed likely that targeting the immigrant population with MDA would be a useful additional measure to add to those already implemented since 2011. The MDA intervention was approved by the Working Group on Vector-borne Diseases of the Hellenic Center for Disease Control and Prevention (HCDCP) and the National Committee for the Management of Tropical Diseases of the Greek Ministry of Health. The project was placed under the “Integrated surveillance and control programme for West Nile virus and malaria in Greece (MALWEST)”. Herein are described the details of this intervention and its overall impact.

## Materials and Methods

### Intervention area characteristics and target population

The antimalarial MDA program was implemented in the agricultural area of Evrotas, Laconia in southern Greece, epicenter of the 2011 outbreak. The particular area is favourable for the expansion of anopheline population [17]; freshwater springs, a complex network of 130 km of irrigation and drainage canals, the Evrotas river delta, the brackish Vivari Lake that seasonally dries out, and coastal wetlands. In the Greek temperate climate, *Anopheles* abundance increases during May to October, period when transmission is most likely to occur. Indeed, symptoms onset for the vast majority of locally acquired cases during 2009–2012 in Greece occurred between May and October, with the peak of the 2011 outbreak in Evrotas occurring in September [1]. Thus, malaria transmission in Greece and specifically in Evrotas is considered to be highly seasonal.

Evrotas, an established farming area with large-scale production of oranges and olive oil, attracts many immigrants who reside there to work as farm laborers. These immigrants gradually move in before the summer and remain there until the mid-autumn. The target group for the MDA was the total population of immigrants originating from malaria endemic countries (mainly Pakistan, Afghanistan, and Bangladesh), who resided and worked in the particular area of Evrotas. It should be noted that all immigrants living in Evrotas were young male adults. Thirteen settlements within the Evrotas municipality were included in the MDA program. Starting from September 2011, all immigrants from endemic countries that resided and worked in the Evrotas region were recorded during ACD in a database developed by the MALWEST project, using an internal coding system for the immigrant residences and all individuals. The immigrants’ registry was developed by conducting house-to-house visits for fever screening and with mediators from the immigrant community. In 2013, all immigrants were asked to participate in the MDA intervention, with the assistance of a mediator after explaining the treatment rationale and possible side effects. In 2014 MDA was delivered only to new immigrants in the area, and to those who for any reason had not received therapy or who received incomplete treatment in 2013. The field teams prioritized the immigrant residences that were closest to mosquito breeding sites. The target population (immigrants) in the Municipality of Evrotas represented almost 10% of the total population (~11,000 including immigrants).

### Treatment protocol

The combination of chloroquine plus primaquine was selected as the drug regimen of choice for this intervention. This regimen is recommended by national (HCDCP) as well as international (WHO and Centers for Disease Control and Prevention, CDC) guidelines [18,19]. Chloroquine tablets were administered at all participants in the form of bisphosphate salts at an initial dose of 1000mg (620mg base) followed by 500mg at 6, 24 and 48 hours. Primaquine tablets were administered for 14 days at a dose of 30mg base per day, after testing of levels of glucose-6-phosphate dehydrogenase (G6PD), using a quantitative enzymatic colorimetric method (L&D DIAGNOSTICS LTD), in view of the potential of primaquine to precipitate hemolytic reactions. Immigrants with moderate or severe deficiency were not given any therapy, while those with mild deficiency received chloroquine as above and a modified regimen of 45mg of primaquine once a week for eight weeks [18]. Directly Observed Treatment (DOT) was used for all participants except for those who left the area of the study before completing the regimen. For these immigrants Supervised Observed Therapy (SOT) was applied through daily telephone communication with them [20]. Only one round of mass treatment was administered over the course of the transmission period. Four field teams, each comprising 1–2 health professionals and a mediator, implemented drug administration. The medications were delivered between 19:00–22:00 when immigrants returned from work for dinner. All drugs were taken with food to minimize gastrointestinal side effects [21]. All adverse effects were systematically recorded in structured pharmacovigilance forms, and were promptly treated by the health professionals from the field team. If necessary, the immigrant was referred for further care to the local hospital. For every individual receiving medication in the context of this intervention, the field team was required to complete a ‘daily malaria regimen observation sheet’ (a copy was provided to the recipients at the end of the regimen), where the day and time of every drug dose were recorded along with the health worker delivering the drug, and any eventual adverse reactions.

### Timing

As appropriate time for implementation of MDA was considered the period before the onset of peak adult mosquito vector activity [8,13,22], which occurs in July and August in Evrotas. Thus, the field team deployed MDA to the immigrant workers target population prior to July, and continued providing one prophylactic antimalarial regimen and to any new immigrants entering the area until end of November. In 2013, MDA started on 22. May and ended on 21. November, while in 2014, MDA started on 28. June and ended on 30. November.

### Other malaria control measures

ACD was initiated in October 2011 in Evrotas for early detection and treatment of malaria cases with the aim to interrupt the malaria transmission in the area. ACD included frequent house visits for fever screening in lodgings where immigrants from endemic countries resided. ACD was implemented in all areas in Greece where locally acquired malaria cases were recorded in 2011 and 2012, as well as areas considered being of high risk because of the presence of the mosquito vector and an inflow of potential parasite carriers (immigrants from endemic counties). Fever screening visits in the context of ACD were performed on a weekly basis for the target population from 2011 to2014. Visits to all immigrant residences were supported by a geographic information system (GIS) application. The implementation of ACD was further supported by creating and using specific standardized forms: immigrant’s registry form, follow-up forms, fever screening forms etc.

During these visits, a rapid diagnostic test (RDT) for malaria was performed for all persons with fever (defined as temperature >37.0°C) or who reported fever and/or other malaria compatible symptoms during the previous week and blood was drawn for blood smear and molecular diagnosis of malaria. Prompt and adequate supervised treatment (chloroquine and 14-day primaquine) was provided to all persons who tested positive.

Immediate case investigation was carried out in order to classify the malaria case as imported or locally acquired with a structured case investigation form. In addition, a focus investigation was conducted in aradius of 100 meters around the residence of the locally acquired case, including all residents (Greek and immigrants). All the residents within this area were informed about malaria signs and symptoms, were investigated for any malaria symptoms and followed up for a total of four weeks on a weekly basis.

Vector control, an integral part of the malaria elimination program deployed in Evrotas, mainly consisted of an integrated mosquito control program financed by the local authorities along with Indoor Residual Spraying (IRS) and the provision of Long Lasting Insecticide-treated Nets (LLINs). IRS was implemented in Evrotas for three years (2012–2014). It mainly took place in two rounds, with an intermediate of 2–3 months, in 150–200 residences of both Greeks and immigrants. The selection of residences for the IRS was based on the proximity to mosquito breeding sites. Evaluation of the residual activity of insecticides was assessed every 30 days for five months until November 2013–2014 through contact bioassays. Colorimetric tests were also used in parallel to the analytic methods to accelerate the results and improve the vector control activities. On the other hand, mosquito control was based on larvicidal applications through private contractors and aimed to eliminate potential anopheline breeding sites. The major breeding sites selected for larvicidal applications included Vivari Lake, coastal reed beds, the old river bed of river Evrotas, river Vasilopotamos, as well as draining canals. Temporary mosquito breeding sites were also detected either within the agro-ecosystems or in small uncontrolled landfills (dumps). In order to evaluate the effectiveness of the larvicidal applications the population density of aquatic and adult mosquito stages was estimated. When increased anopheline population densities were recorded, supplementary interventions, such as aerial sprayings over extensive water bodies with difficult access (for example, Vivari Lake), were performed. During transmission periods 2013–2014, LLINs were also distributed to the majority of the immigrants in the area. The field team delivered the nets to the leader of each residence, ensuring their correct installation and use. During the ACD and MDA visits, the field team continued to check and correct any discrepancies of net use.

Enhanced laboratory capacity in malaria diagnosis and the introduction of RDT in primary health care settings [23] were two primary goals achieved through continuing education and training seminars for laboratory personnel and health professionals. Personnel from the National School of Public Health (NSPH) conducted seminars in local health care facilities, while educational sessions performed by HCDCP personnel took place in many villages (mainly attended by local population) as an effort to gain local population trust and cooperation.

### Immigrant worker community engagement

The field teams built considerable trust with the immigrant community through their work in the context of ACD and delivery of basic primary care in 2011 and 2012. Extensive and repeated efforts were made to ensure that the purpose of the program and its expected outcome were clearly understood by the immigrants, and that their collaboration and contribution to its success were crucial. Accurate and frequent communication was secured via the ACD visits and by the presence of mediators from the immigrant community.

### Costs of the MDA intervention

All the costs associated with this intervention, including health staff (doctors and nurses), mediators, costs for laboratory testing (G6PD levels), procurement of pharmaceuticals (chloroquine and primaquine), treatment of side effects, consumables, as well as transportation cost, were covered by the Greek Ministry of Health and the HCDCP. It should be noted that medication as well as primary care services during MDA and ACD were provided free of charge to the immigrant workers.

### Ethics statement

The MDA in Evrotas was approved by the Committee for Vector-Borne Diseases in the Hellenic Center for Disease Control and Prevention (HCDCP) and by the Review Board of the Greek Ministry of Health. All participants were male adults and informed of the rationale, methodology and expected outcomes of the MDA intervention. The procedures, potential risks-side effects of drugs and benefits were verbally explained in detail to all participants. Written consent statement in their language was completed by all participants. Moreover, mediators/translators participated in all the interactions with the immigrants and they supported the process of signing the written consent statements.

## Results

**Fig 2:**
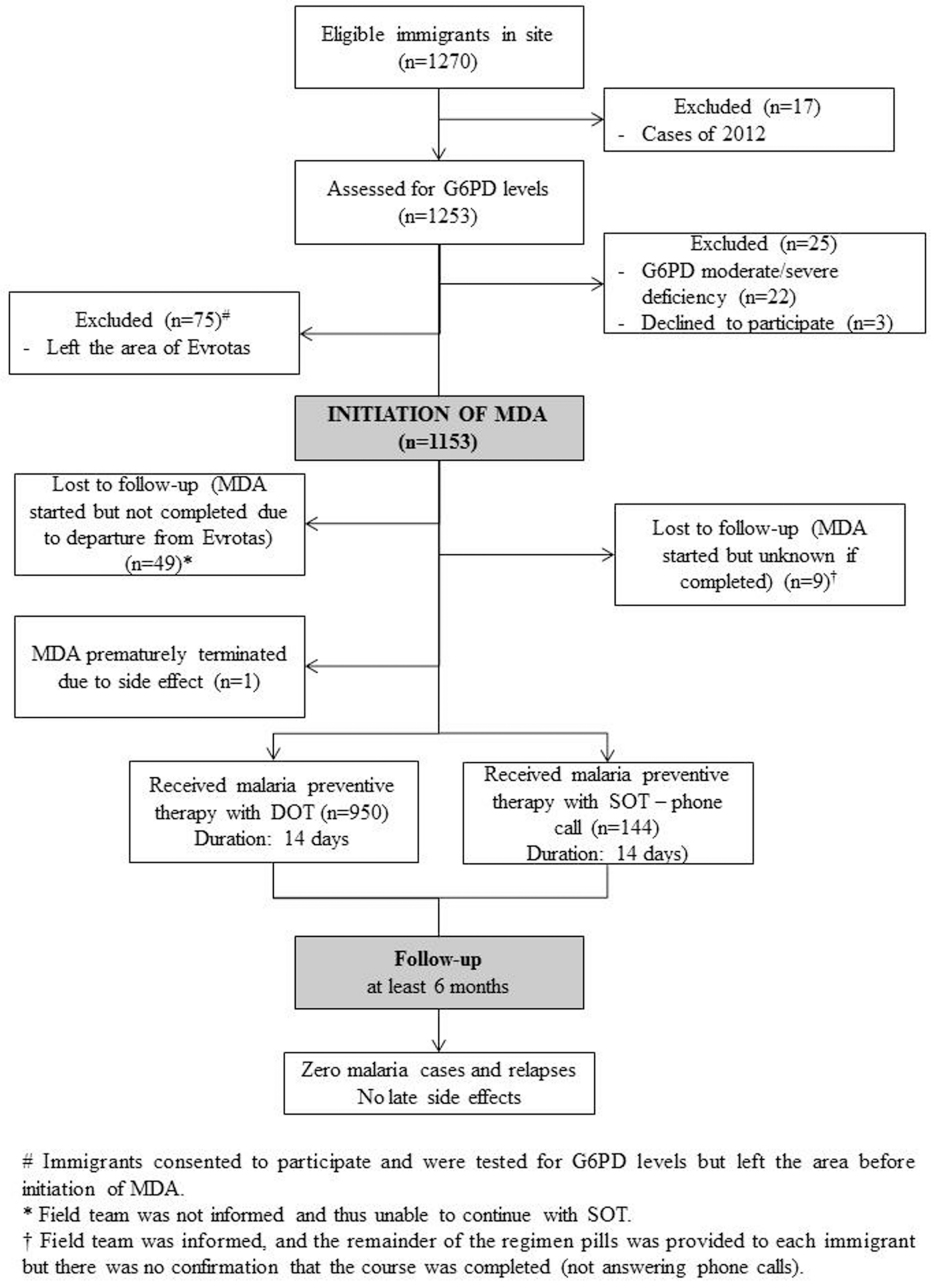
MDA flowchart during the years 2013 and 2014 in the area of Evrotas, Greece.

**Fig 3:**
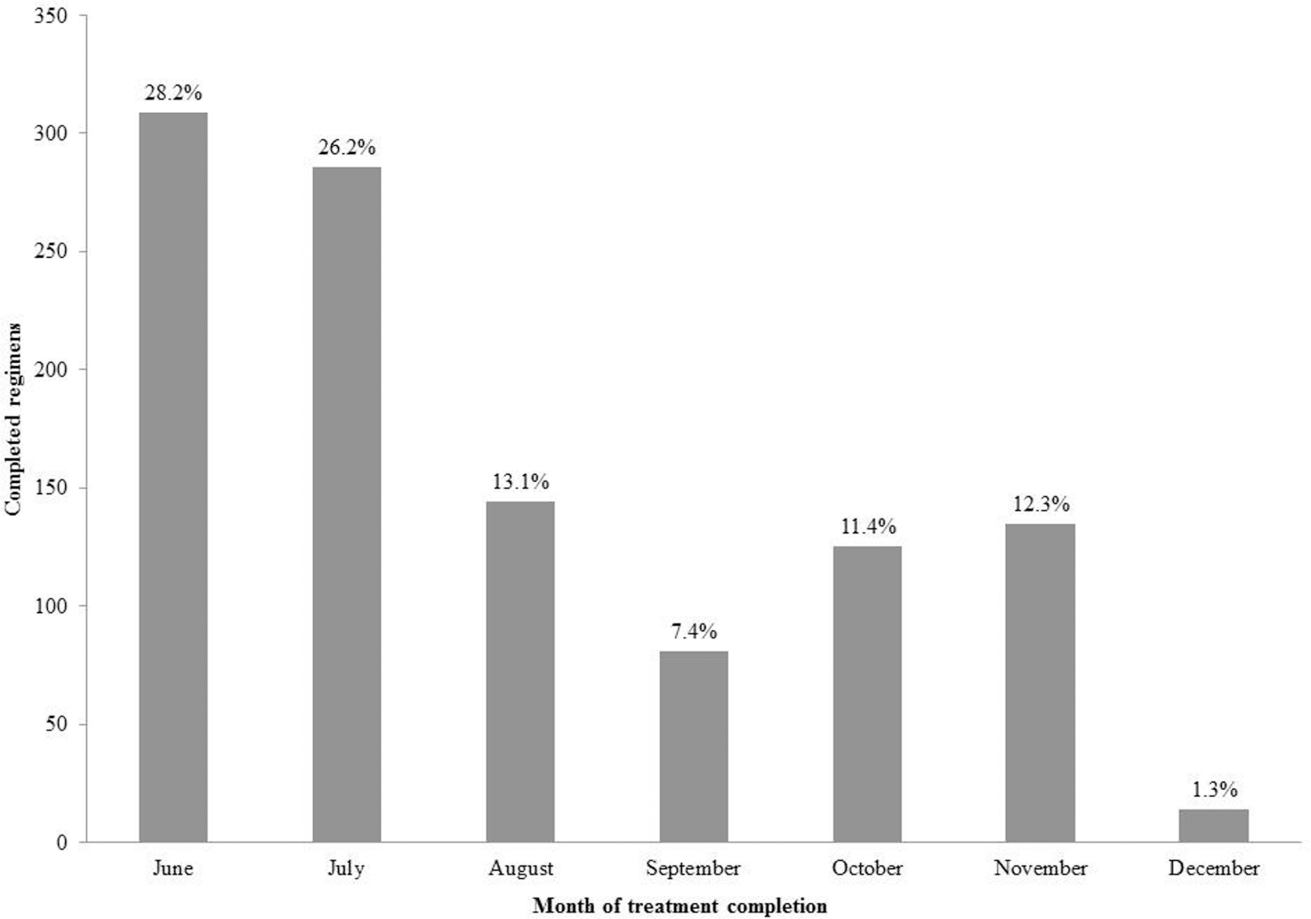
Monthly distribution (number and percentage) of the completed regimens in 1,094 immigrants in 2013 and 2014 in Evrotas, Greece.

One thousand two hundred seventy (1270) immigrants were eligible to participate in the intervention in the years 2013 and 2014. Seventeen (17) immigrants, who had been diagnosed with malaria in the 2012 transmission period and had received therapy, were excluded. A total of 1094 immigrants from malaria-endemic countries received MDA during the transmission periods 2013 and 2014 (862 in 2013 and 232 in 2014). The vast majority of immigrants were from Pakistan or Afghanistan (Table 1), with a smaller number from Bangladesh and other Asian countries. The immigrants often resided outside settlements close to mosquito breeding sites in poor housing conditions, principally sheds, outhouses and other makeshift housing without proper doors or windows to reduce man-mosquito contact. The immigrant target population was not stable during the transmission period, with significant turnover where about 30% moved in or out of the area depending on work availability. All eligible immigrants were initially checked for G6PD, while 75 of them left the area despite having initially agreed to participate in the MDA intervention and they were excluded. Testing for G6PD levels identified 22 immigrants with moderate or severe deficiency, who were also excluded. The majority of the target population (950, 75.8%) underwent DOT with the antimalarial regimen as described above, while the remaining 144 received SOT (Fig 2), bringing the total treatment coverage to 87.3% of the target population. Initiation and completion of treatments before the end of July and the peak of the transmission period was ensured for the majority of immigrants who were present in the particular area, while for the newly arriving immigrants the treatment was initiated within two weeks of their arrival. The monthly distribution of completed treatments is shown in Fig 3.

**Table 1:** Characteristics of the target immigrant population at Evrotas (n = 1094), for 2013 and 2014.

Immigrants characteristics		Number	Percentage
**Male gender**		1094	100%
**Age**		Range 18–63 (median: 28.5)	N/A
**Country of origin**	Pakistan	952	87%
	Bangladesh	58	5.3%
	Afghanistan	75	6.8%
	India	3	0.3%
	Iran	1	0.1%
	Unknown	5	0.5%
**Education (mean years in school)**		6.9	N/A
**Duration of stay in Greece (months, median)**		38.3	N/A

Adverse events associated to primaquine administration were only reported by 145 (13.2%) of individuals, with gastrointestinal symptoms as the predominant complaint. Nearly one third of the participating immigrants (393, 35.9%) mentioned one or more side effects associated with chloroquine administration, such as headaches, dizziness or gastrointestinal symptoms (Table 2). A single potentially serious case of hemolysis was recorded throughout the MDA program in one immigrant on the second dose of the 14-day primaquine course in 2013. The patient had clinical signs of hemolysis and upon admission to the local hospital; blood count revealed 20% decrease of hematocrit, increase of creatinine (2.5 mg/dL), and total bilirubin (9.26 mg/dL) with indirect Coombs test negative. The immigrant fully recovered after a few days of hospitalisation. Prior to enrolment his G6PD test had indicated normal values (G6PD: 7.2 U/g Hb), but independent tests conducted two months after the hemolytic episode indicated moderate to severe deficiency (G6PD: 3.4 U/g Hb from one laboratory, and G6PD: 0.7 U/g Hb from another). This outcome could be related to the accuracy of the specific G6PD laboratory test or the patient had in the past hemolytic anemia due to malaria relapse or for unknown reasons. All the other adverse events recorded were minor and resolved following simple medical advice or with supportive symptomatic care.

**Table 2:** Reported adverse events associated to chloroquine and primaquine administration (n = 1094), for 2013 and 2014.

Side effect/Symptom	Chloroquine	95% C.I.	Primaquine	95% C.I.
	No of events		No of events	
**Dizziness**	143 (13.1%)	11.2–15.2	0	
**Headache**	141 (12.9%)	11.0–15.1	0	
**Diarrhoea**	50 (4.6%)	3.4–6.0	0	
**Abdominal pain** *	76 (6.9%)	5.5–8.7	85 (7.8%)	6.3–9.6
**Nausea/vomiting** *	67 (6.1%)	4.8–7.8	67 (6.1%)	4.8–7.8
**Muscular weakness**	11 (1.0%)	0.5–1.8	0	
**Sleep disturbance**	18 (1.6%)	1.0–2.6	0	
**Skin rash**	13 (1.2%)	0.7–2.1	0	
**Vision disorders**	7 (0.6%)	0.3–1.4	0	
**Photosensitivity**	2 (0.2%)	0–0.7	0	
**Anorexia** *	2 (0.2%)	0–0.7	5 (0.5%)	0.2–1.1
**Hemolytic anaemia**	0		1 (0.1%)	0–0.6

* Common symptoms for both medications. The first 48 hours of co-administration, symptoms were attributed to either drug based on clinical judgement at the field.

Preliminary data from the cost analysis of this intervention in 2013 and 2014 indicate a total cost of about 112,000 Euros. The cost for laboratory testing and drug procurement was 8,000 Euros, while cost of transportation 18,000 Euros. Staff cost amounted to more than 85,000 Euros, 75% of the total cost. Thus, an expenditure of a little more than 88 Euros was incurred for each of the 1270 immigrants that were eligible for preventive therapy in transmission periods 2013 and 2014.

As shown in Table 3, no cases of malaria, imported or locally acquired, were recorded for 2013 or 2014, which suggests that the outbreak noted in Evrotas in the previous years was at an end.

**Table 3:** Reported malaria cases by year of onset and case classification, Evrotas and other regions in Greece, 2009–2014.

	Evrotas		Other regions in Greece	
Year of symptom onset	Locally acquired	Imported	Locally acquired	Imported
**2009**	6	0	1	44
**2010**	1	0	3	40
**2011**	36	21	6	33
**2012**	10	17	10	56
**2013**	0	0	3	22
**2014**	0	0	0	38

## Discussion

This article describes the first mass administration of antimalarial treatment in Greece several decades after the malaria elimination programs that led to the declaration of the country as malaria free in 1974. The choice to deploy an MDA scheme in Evrotas was dictated by the failure of the extensive control measures implemented in 2011 and 2012 to stem the occurrence of locally acquired malaria in the 2012 transmission period. It is highly likely that the implementation of the MDA scheme led to the sustainable interruption of transmission in 2013 and 2014, during which no malaria cases were recorded in Evrotas.

The recent adoption by WHO of elimination as the ultimate goal of malaria control programs worldwide has revived interest in MDA as a potential means to achieve this. Evidence for the efficacy of MDA in the context of malaria is limited to favourable short-term outcomes [8,24,25], with sustainable outcomes more likely in low transmission settings in geographically restricted areas (for example, islands) [7,11,26,27]. The main concerns in advocating MDA are the costs needed to achieve high coverage and the logistical support, the need for a safe drug regimen, and the possibility to minimize the risk of promoting drug resistance.

A number of factors made the Evrotas area suitable as for MDA deployment. The malaria outbreaks in Evrotas were confined to a small geographical area in an otherwise malaria-free country, where the transmission period is seasonal and relatively short. Furthermore, the target population was relatively small in number, well defined (immigrants from the Indian sub-continent), and resided in limited locations easily accessible to health workers, thereby lightening the logistical burden leading to a high rate of coverage [28]. It was deemed important to sensitize and gain the confidence of the target population before initiating the MDA program [29], a task that was achieved by the field teams who also ensured that aims of the treatment and the consent procedures were fully understood [30]. In this manner, nearly 90% of the target population in Evrotas successfully received the full treatment.

It was decided to administer both chloroquine and primaquine since the parasite species diagnosed in this area was *P*. *vivax*. Thus, the immigrants who were detected with malaria were probably suffering from a relapse episode, and the parasite reservoir was predominantly present as hypnozoites in the liver (making any attempts to restrict treatment to infected individuals superfluous). This is consistent with the relapse timing of the long latency *P*. *vivax* strains circulating in the countries of origin of the majority of the target group, Pakistan and Afghanistan [31,32]. The combination of the two drugs helped minimize the risk to select drug resistance [11,33]. Treatment of *P*. *vivax* cases in Greece is supervised by a follow-up protocol, with microscopic examination conducted weekly until day 28 post-treatment and with a polymerase chain reaction (PCR)-based detection assay carried out on samples collected on days 14 and 28. Recurrent parasitemias have not been observed, correlating appropriately with the lack of evidence of widespread resistance to chloroquine or primaquine in *P*. *vivax* from the sub-Indian continent [34–36]. Even in the event of low grade chloroquine resistance, primaquine coadministration would cover the gap as primaquine has activity against blood and liver stages, including against chloroquine-resistant strains [36]. Thus, it would seem unlikely that the MDA in Evrotas would select for drug resistant strains, especially as primaquine remains active against the blood and liver stages of chloroquine-resistant strains.

Drug safety is of paramount importance in MDA, as only a minor proportion of the target population, those carrying the parasite, will benefit at the individual level [33]. Thus, active surveillance and careful recording of side-effects were implemented for the MDA intervention in Evrotas. Health professionals strictly implemented DOT throughout the MDA program in Evrotas. DOT, though costly, is considered essential to securing high coverage and for effective monitoring of potential treatment complications [28]. The vast majority (86.8%) of immigrants receiving primaquine did not experience any serious side effects, with abdominal pain and nausea/vomiting as the most common minor complaints reported, as in previous studies [37,38]. The single case of hemolysis recorded in an immigrant from Pakistan was due to a false normal initial laboratory result, however, the close daily surveillance of potential adverse effects led to prompt management and a full and uneventful recovery. As far as chloroquine is concerned the commonest side effects were dizziness and headache, which were often reported as side effects of the drug in previous studies as well [16,39]. The side effects due to chloroquine were more frequent than those due to primaquine, but all were minor and none resulted in interrupting the treatment.

From an operational point-of view, it was not feasible to administer antimalarial drugs simultaneously to all the immigrants. The target population was not stable, with people constantly moving in and out of the area. Moreover, this would have required a large number of field workers to ensure adequate monitoring of drug distribution and administration and the associated ACD visits and daily follow-up medical supervision to ensure prompt action in case of adverse events. Priority in the initiation of MDA was given to immigrants living in close proximity to *Anopheles* breeding sites. In the context of the highly seasonal nature of transmission in Evrotas, a single round of MDA was deemed sufficient [22]. The MDA delivery was initiated as soon as the immigrant workers started to move into the area in early summer, a little after the start of the transmission season (late spring), but MDA was completed before the peak transmission period of July-August. For those newly arriving to Evrotas thereafter recruited for treatment at most within two weeks of arrival. Ultimately, long-term sustainability is an important end-point for MDA. A recent review of the literature identified 12 MDA studies demonstrating zero locally acquired malaria cases in the target population over the six months following drug administration [28]. In Evrotas, no cases of malaria were recorded during the 22 months following the first MDA program, while in the greater area of Laconia two imported cases were recorded; one in an immigrant, and one in a traveller returning from an endemic area. Classic malaria control measures, such as ACD and appropriate vector control, should remain in place to maintain this full interruption of transmission. It should be noted that ACD proved to be a useful tool for early detection and treatment of malaria cases in 2011 and 2012 since 17 cases out of 27 (62.9%) were actively detected. Full details of the ACD intervention are going to be published in a follow-up paper.

Although a formal cost analysis could not be made, an estimation of the costs associated mainly to the MDA intervention indicates that about the cost was about 88 Euros per individual. Given that this contributed to achieving an interruption of malaria transmission, this sum can be considered to be cost-effective when one considers the direct and indirect economic, social and politic burdens that would have been imposed by a sustained reintroduction of malaria in Greece, and the avoidance of Greece being certified as an endemic country.

We are aware that the MDA in Evrotas is an observational study, lacking a control group that was implemented in parallel with on-going classical control measures. This makes it difficult to assess the extent to which the MDA contributed to the absence of malaria cases in the two years following its initiation. Given that the control measures implemented in the previous two transmission periods failed to fully prevent local transmission, it seemed reasonable to ascribe a significant role of MDA in preventing further cases in Evrotas. Whereas the number of imported malaria cases in Greece has been relatively constant since 2009 until 2012, sustained local transmission seemed to have become established only in Evrotas Municipality (53 of the 73 locally acquired cases recorded in Greece for the 2009–2012 period).Moreover, the locally acquired cases in 2011 in Evrotas were more than those recorded among immigrants probably due to malaria underdiagnosis among immigrants since the ACD started in October 2011 and some immigrants (possible cases) left the area before the ACD initiation. According to published data, microsatellite analysis indicated that some of the cases recorded in the immigrants in Evrotas might have actually been locally acquired [9]. Thus, the area of Evrotas could be considered highly vulnerable for malaria, while it is not known whether this is due to environmental conditions specific to Evrotas, to an origin of the immigrant population that moved there from a particularly malarious native area, or a combination of both. Outside Evrotas, the locally acquired cases were distributed across widespread geographical locations throughout Greece. The fact that only 3 locally transmitted cases were observed in the rest of Greece in 2013, and none in 2014, probably reflects the heightened awareness of malaria by public health authorities together with the decrease of the immigrants’ influx from endemic countries [40]. It is clearly important to monitor malaria cases with renewed vigilance and to ensure rapid implementation of appropriate control measures to prevent local transmission.

The reintroduction of vivax malaria in Greece is a possible threat that needs to be confronted. The inflow of large numbers of immigrants from malaria endemic areas, especially from those where *P*. *vivax* occurs, constituted a reservoir of carriers resident in an area where the presence of an efficient malaria vector associated with permissive environmental and social conditions led to locally acquired malaria transmission nearly 40 years after its interruption. Such conditions pose the serious possibility of reintroducing malaria in other regions of Greece or indeed in other countries with similar settings, a threat that must be countered early and effectively. The adoption of a preventive MDA scheme appeared to have made a crucial addition to the control measures initially deployed in the Evrotas area in that no further cases of malaria were recorded over two years since its implementation. In addition to the important operational experience that was gained in carrying out an effective and safe MDA scheme, this episode suggests that MDA tailored to each local epidemiological setting should be considered seriously as a potential strategy, complementary to other antimalarial measures, to hasten, achieve and maintain malaria elimination.
